# Supplementary material for: Prevalence and risk factors of disordered eating risk among Yemeni medical students: A national multicenter cross-sectional study
Source: PLoS One. 2026 Mar 25;21(3):e0345771. doi: 10.1371/journal.pone.0345771 (PMC13016294; doi:10.1371/journal.pone.0345771)
Supplement: Attachment — Submitted filename: PONE-D-25-61542-Final_reviewer.pdf [file pone.0345771.s003.pdf]

PLOS ONE

Uncovering the Burden and Risk Factors of Eating Disorders Among Yemeni Medical Students: A National Multicenter Cross-Sectional Analysis

--Manuscript Draft--

**Manuscript Number:**	PONE-D-25-61542
**Article Type:**	Research Article
**Full Title:**	Uncovering the Burden and Risk Factors of Eating Disorders Among Yemeni Medical Students: A National Multicenter Cross-Sectional Analysis
**Short Title:**	Eating Disorders Among Yemeni Medical Students
**Corresponding Author:**	Ehab sharyan<br>Sana'a University Faculty of Medicine<br>sanaa, YEMEN
**Keywords:**	Eating disorders; medical students; Yemen; EAT-26; Disordered eating behaviors
**Abstract:**	Purpose<br><br>This study aimed to screen for the risk of eating disorders among medical students in Yemen and to identify associated sociodemographic, clinical, and psychological factors.<br><br>Methods<br><br>A descriptive cross-sectional study was conducted from February to June 2025 among undergraduate medical students enrolled in universities in Yemen. Participants completed a self-administered online questionnaire including the Eating Attitudes Test-26 (EAT-26), a validated screening tool for ED risk, along with items assessing sociodemographic characteristics, lifestyle factors, mental health history, and previous ED diagnoses. Data were analyzed using SPSS version 28.0. Descriptive statistics summarized participant characteristics, and logistic regression was used to identify factors associated with disordered eating symptoms.<br><br>Results<br><br>A total of 3,540 students participated in the study. 30.4% (n = 1,076) screened positive for disordered eating behaviors (EAT-26 score ≥ 20). Additionally, 31.6% of participants reported a previous diagnosis of an ED. Key risk factors identified in multivariate analysis included food insecurity, psychological comorbidities, history of suicide attempts, and parental perception of the participant as overweight (all p < 0.001). Protective factors included regular physical activity and a history of Type 1 Diabetes Mellitus (T1DM) or autism. Behavioral symptoms such as binge eating (reported by 40.1%), induced vomiting (27.5%), and use of weight control substances (27.6%) were prevalent, with a notable proportion engaging in these behaviors frequently. Body weight dissatisfaction and perfectionistic or impulsive personality traits were also significantly associated with increased ED risk.<br><br>Conclusion<br><br>Disordered eating behaviors are prevalent among Yemeni medical students, with nearly one-third at risk based on EAT-26 scores. The findings highlight the urgent need for early screening, increased awareness, and targeted interventions within this population. Given the range of psychological and social risk factors identified, future efforts should incorporate mental health support, nutritional education, and culturally sensitive prevention strategies.
**Order of Authors:**	Naji Al-bawah
	Mohamed Baklola
	Anas H. Khalifeh

Powered by Editorial Manager® and ProduXion Manager® from Aries Systems Corporation

	Ehab sharyan
	Ahmed Al-Eryani
	Anas Zakarya Nourelden
	Saif Alaribi
	Fayda Al-wesabi
	Aaida Al wesabi
	Ahdab Al-Jaberi
	Sama Shamsan
	Ahmed Abdulmughni
	Ghailan Al-Jarbani
	Hisham Salman
	Amira Yasmine Benmelouka

Additional Information:	
**Question**	**Response**
**Financial Disclosure**<br><br>Enter a financial disclosure statement that describes the sources of funding for the work included in this submission. Review the submission guidelines for detailed requirements. View published research articles from *PLOS ONE* for specific examples.<br><br>This statement is required for submission and **will appear in the published article** if the submission is accepted. Please make sure it is accurate.<br><br>**Funded studies**<br>Enter a statement with the following details:<br>• Initials of the authors who received each award<br>• Grant numbers awarded to each author<br>• The full name of each funder<br>• URL of each funder website<br>• Did the sponsors or funders play any role in the study design, data collection and analysis, decision to publish, or preparation of the manuscript?<br><br>Did you receive funding for this work?	The author(s) received no specific funding for this work.
**Competing Interests**	The authors have declared that no competing interests exist

Use the instructions below to enter a competing interest statement for this submission. On behalf of all authors, disclose any competing interests that could be perceived to bias this work—acknowledging all financial support and any other relevant financial or non-financial competing interests.<br><br>This statement is required for submission and **will appear in the published article** if the submission is accepted. Please make sure it is accurate and that any funding sources listed in your Funding Information later in the submission form are also declared in your Financial Disclosure statement.<br><br>View published research articles from *PLOS ONE* for specific examples.<br><br>**NO authors have competing interests**<br><br>Enter: *The authors have declared that no competing interests exist.*<br><br>**Authors with competing interests**<br><br>Enter competing interest details beginning with this statement:<br><br>*I have read the journal's policy and the authors of this manuscript have the following competing interests: [insert competing interests here]*<br><br>* typeset	
**Ethics Statement**<br><br>Enter an ethics statement for this submission. This statement is required if the study involved:<br><br>• Human participants<br>• Human specimens or tissue<br>• Vertebrate animals or cephalopods<br>• Vertebrate embryos or tissues<br>• Field research	N/A

Write "N/A" if the submission does not require an ethics statement.

General guidance is provided below. Consult the submission guidelines for detailed instructions. **Make sure that all information entered here is included in the Methods section of the manuscript.**

Format for specific study types

Human Subject Research (involving human participants and/or tissue)

- Give the name of the institutional review board or ethics committee that approved the study
- Include the approval number and/or a statement indicating approval of this research
- Indicate the form of consent obtained (written/oral) or the reason that consent was not obtained (e.g. the data were analyzed anonymously)

Animal Research (involving vertebrate animals, embryos or tissues)

- Provide the name of the Institutional Animal Care and Use Committee (IACUC) or other relevant ethics board that reviewed the study protocol, and indicate whether they approved this research or granted a formal waiver of ethical approval
- Include an approval number if one was obtained
- If the study involved *non-human primates*, add *additional details* about animal welfare and steps taken to ameliorate suffering
- If anesthesia, euthanasia, or any kind of animal sacrifice is part of the study, include briefly which substances and/or methods were applied

Field Research

Include the following details if this study involves the collection of plant, animal, or other materials from a natural setting:

- Field permit number
- Name of the institution or relevant body that granted permission

**Data Availability**<br><br>Authors are required to make all data underlying the findings described fully available, without restriction, and from the time of publication. PLOS allows rare exceptions to address legal and ethical concerns. See the PLOS Data Policy and FAQ for detailed information.<br><br>A Data Availability Statement describing where the data can be found is required at submission. Your answers to this question constitute the Data Availability Statement and **will be published in the article**, if accepted.<br><br>**Important:** Stating 'data available on request from the author' is not sufficient. If your data are only available upon request, select 'No' for the first question and explain your exceptional situation in the text box.<br><br>Do the authors confirm that all data underlying the findings described in their manuscript are fully available without restriction?	Yes - all data are fully available without restriction
**Describe where the data may be found in full sentences. If you are copying our sample text, replace any instances of XXX with the appropriate details.**<br><br>• If the data are **held or will be held in a public repository**, include URLs, accession numbers or DOIs. If this information will only be available after acceptance, indicate this by ticking the box below. For example: *All XXX files are available from the XXX database (accession number(s) XXX, XXX.*).<br>• If the data are all contained **within the manuscript and/or Supporting Information files**, enter the following: *All relevant data are within the manuscript and its Supporting Information files.*<br>• If neither of these applies but you are able to provide **details of access elsewhere**, with or without limitations, please do so. For example:	All relevant data are within the manuscript and its Supporting Information files

Data cannot be shared publicly because of [XXX]. Data are available from the XXX Institutional Data Access / Ethics Committee (contact via XXX) for researchers who meet the criteria for access to confidential data.<br><br>*The data underlying the results presented in the study are available from (include the name of the third party and contact information or URL).*<br>• This text is appropriate if the data are owned by a third party and authors do not have permission to share the data.<br><br>* typeset	
Additional data availability information:	

Click here to access/download;Manuscript;4566666666666666666.docx

Uncovering the Burden and Risk Factors of Eating Disorders Among Yemeni Medical Students: A National Multicenter Cross-Sectional Analysis

Naji Al-bawah[1*†], Mohamed Baklola[2*†], Anas H. Khalifeh[3], Ahmed Al-Eryani [4], Anas Zakarya Nourelden [5], Saif Alaribi [6], Ehab sharyan[7], Fayda Al-wesabi[8], Aaida Al wesabi [9], Ahdab Al-Jaberi[10], Sama Shamsan[11], Ahmed Abdulmughni[12], Ghailan Al-Jarbani[13], Hisham Salman[14], Amira Yasmine Benmelouka[15]

1. Faculty of medicine, Sana'a university, Sana'a, Yemen

(Najialbawah@gmail.com)

2. Faculty of Medicine, Mansoura University, 60El-Gomhoria Street, Mansoura 35516, Egypt.

(Mohamedbaklola2000@gmail.com)

3. Department of Community & Mental Health Nursing, Faculty of Nursing, Zarqa University, P.O.Box 132222, Zarqa 13132, Jordan

(akhalifeh@zu.edu.jo)

4. Faculty of medicine, Sana'a university, Sana'a, Yemen

(Al.eryani444@gmail.com)

5. Faculty of Medicine, Al-Azhar University, Cairo, Egypt

(anas.zakarya25@gmail.com)

6. Faculty of Medicine, Emirates International University, Sana'a, Yemen

(Saifnaji931@gmail.com)

7. Faculty of medicine, Sana'a university, Sana'a, Yemen

(Ehab52sharyan@gmail.com)

8. Faculty of medicine,21 September university, Sana'a, Yemen

(pdifferent54@gmail.com)

9. Faculty of Medicine, 21 September University, Sana'a, Yemen

(Professionaldoctor572@gmail.com)

10. Faculty of medicine, sana'a university, sana'a, Yemen

(ahdabali2005@gmail.com)

11. Faculty of Medicine, Sana’a University, Sana’a, Yemen

(samashamsan464@gmail.com)

12. Faculty of medicine, Sana'a university, Sana'a, Yemen

(dr.ahmedakram2003@gmail.com)

13. Faculty of medicine, Sana'a university, Sana'a, Yemen

(aljrbanyg@gmail.com)

14. Faculty of medicine, Sana'a university, Sana'a, Yemen

(Hishamsalman2034@gmail.com)

15. Faculty of medicine, university of Algiers, Algiers, Algeria

(amira.yasmine.benmelouka@gmail.com)

† Naji Al-bawah and Mohamed Baklola contributed equally to this work

Corresponding authors:

Corresponding authors: Mohamed Baklola*, Naji Al-bawah*

E-mails: Mohamedbaklola2000@gmail.com, Najialbawah@gmail.com

Abstract

Purpose: This study aimed to screen for the risk of eating disorders among medical students in Yemen and to identify associated sociodemographic, clinical, and psychological factors.

Methods: A descriptive cross-sectional study was conducted in Yemen, following ethical approval, which was granted on June 21, 2025. The recruitment and data collection for this study began on June 22, 2025, and was completed on July 22, 2025.among undergraduate medical students enrolled in universities in Yemen. Participants completed a self-administered online questionnaire including the Eating Attitudes Test-26 (EAT-26), a validated screening tool for ED risk, along with items assessing sociodemographic characteristics, lifestyle factors, mental health history, and previous ED diagnoses. Data were analyzed using SPSS version 28.0. Descriptive statistics summarized participant characteristics, and logistic regression was used to identify factors associated with disordered eating symptoms.

Results: A total of 3,540 students participated in the study. 30.4% (n = 1,076) screened positive for disordered eating behaviors (EAT-26 score ≥ 20). Additionally, 31.6% of participants reported a previous diagnosis of an ED. Key risk factors identified in multivariate analysis included food insecurity, psychological comorbidities, history of suicide attempts, and parental perception of the participant as overweight (all $p < 0.001$). Protective factors included regular physical activity and a history of Type 1 Diabetes Mellitus (T1DM) or autism. Behavioral symptoms such as binge eating (reported by 40.1%), induced vomiting (27.5%), and use of weight control substances (27.6%) were prevalent, with a notable proportion engaging in these behaviors frequently. Body weight dissatisfaction and perfectionistic or impulsive personality traits were also significantly associated with increased ED risk.

Conclusion: Disordered eating behaviors are prevalent among Yemeni medical students, with nearly one-third at risk based on EAT-26 scores. The findings highlight the urgent need for early screening, increased awareness, and targeted interventions within this population. Given the range of psychological and social risk factors identified, future efforts should incorporate mental health support, nutritional education, and culturally sensitive prevention strategies.

Keywords: Eating disorders, Medical students, Yemen, EAT-26, Disordered eating behaviors

Background

Eating disorders (EDs) are complex psychiatric conditions marked by persistent disturbances in eating behaviors and an excessive focus on body weight and shape. These disorders significantly impair mental, physical, and psychosocial functioning(1–3). According to the Diagnostic and Statistical Manual of Mental Disorders, Fifth Edition (DSM-5), EDs encompass a range of conditions, including Anorexia Nervosa (AN), Bulimia Nervosa (BN), Binge Eating Disorder (BED), Avoidant/Restrictive Food Intake Disorder (ARFID), Pica, Rumination Disorder, and Other Specified Feeding or Eating Disorders (OSFED) (4). Disordered eating exists on a continuum, ranging from healthy eating habits to clinically diagnosable EDs, and can result in serious physiological consequences such as electrolyte imbalances, hormonal disruptions, cardiovascular and renal dysfunction, and increased risk of bone fractures (5).

The development of EDs is multifactorial. Predisposing factors include genetic vulnerabilities and certain personality traits, while triggering and maintaining factors may involve environmental influences, social isolation, and maladaptive coping mechanisms (6). Globally, the prevalence of EDs is estimated at 5.7% among females and 2.2% among males (7). However, rates appear higher in specific subgroups, particularly medical students. A recent systematic review and meta-analysis reported a prevalence of 10.4% among medical students (8), while other individual studies suggest rates reaching 15% (9). These figures vary widely by country, with prevalence reported at 2% among Chinese medical students (10), 28.6% in Jordan (11), and specific ED diagnoses such as binge eating and bulimia nervosa reported at 5% and 1.2% respectively, in Nepal (12).

Medical students are considered particularly vulnerable to EDs due to a combination of academic, psychological, and social pressures. Contributing factors may include anxiety, depression, academic overload, burnout, limited coping skills, lack of social support, weight stigma, and body image dissatisfaction (13–17). Studies also suggest that vulnerability may increase during the transition to clinical training years (18). Difficulties in emotional processing, including alexithymia and impaired social cognition, may further elevate this risk (19).

EDs among students are associated with diminished cognitive performance, mood disturbances, decreased motivation, and impaired academic achievement (20). Early identification of

disordered eating patterns is crucial to preventing the escalation to more severe clinical conditions (21). Preventive strategies such as resilience training, mental health counseling, nutrition education, and stigma-reduction programs have shown potential in addressing this issue (9,13,22).

Although interest in eating disorder research has grown in the Middle East and North Africa (MENA) region, significant data gaps remain (23). A recent large-scale multinational study involving over 5,000 medical students across the MENA region reported that approximately 24.8% were at risk of eating disorders, with key risk factors including type 1 diabetes mellitus, schizophrenia, autism, female gender, inflammatory bowel disease (IBD), and frequent exposure to the thin-ideal body image (24). Protective factors included regular physical activity and satisfaction with one’s weight. Despite the breadth of this study, Yemen was not among the countries assessed, and to date, no published studies have specifically examined the risk of eating disorders among Yemeni medical students. Given Yemen's distinct sociocultural dynamics, ongoing conflict, limited mental health resources, and increasing influence of media-driven body ideals, it is critical to investigate the extent and determinants of disordered eating in this population. Therefore, this study aims to assess the risk of eating disorders among medical students in Yemen and ~~to~~ identify the sociodemographic and psychosocial factors associated with increased vulnerability.

Methods

Study design

A descriptive cross-sectional study design with an analytical component was adopted to evaluate the prevalence of disordered eating symptoms and their associated factors among medical students in Yemen. This manuscript was prepared by the Strengthening the Reporting of Observational Studies in Epidemiology (STROBE) checklist for cross-sectional studies. The completed checklist is available in S1 File.

Study population

The target population included undergraduate medical students enrolled in Yemeni universities. Inclusion criteria encompassed students from all academic years (first

through sixth) who voluntarily agreed to participate in the study.

Study setting and duration

The study was conducted across fifteen medical schools in Yemen, following ethical approval, which was granted on June 21, 2025. The recruitment and data collection for this study began on June 22, 2025, and were completed on July 22, 2025. The selected institutions ensured diversity in educational settings and broad geographical representation.

Sample size calculation:

The required sample size was calculated using G*Power software (version 3.1.9.7). An a priori power analysis was conducted for a logistic regression model using a two-tailed test, an odds ratio of 1.3, a significance level (α) of 0.05, and a desired statistical power of 0.95. Assuming a normal distribution of the predictor and a null hypothesis probability ($Pr(Y = 1 \mid X = 1) H_0$) of 0.2, the analysis indicated that a total sample size of 1,188 participants would be required to detect the specified effect. To compensate for a possible 20% non-response rate or incomplete data, the target sample size was increased to 1,426 participants. However, the data collection ultimately yielded a much larger sample size of 3,540 participants, expanding the study's statistical power and robustness.

Participants

Eligible participants were undergraduate medical students enrolled in Yemeni medical colleges at the time of the study. Inclusion criteria were: (1) age of 18 years or older, and (2) current enrollment in any academic year from first to sixth. Students were invited to participate voluntarily, and no incentives were offered.

Instruments

The questionnaire consisted of three sections. The first section gathered socio-demographic and educational information, including age, gender, academic year, and lifestyle-related variables such as self-reported weight, height, and physical activity levels.

The second section assessed disordered eating symptoms using the Eating Attitudes Test (EAT-26), a widely used and validated screening tool for identifying individuals at risk of eating disorders (25). The EAT-26 includes 26 items, each with six response options. For items 1 to 25,

responses of “sometimes,” “rarely,” or “never” were scored as 0, while “often,” “usually,” and “always” were scored as 1, 2, and 3, respectively. Item 26 was reverse scored: responses of “always,” “usually,” or “often” received a score of 0, whereas “sometimes,” “rarely,” and “never” were scored as 1, 2, and 3 respectively. The total score ranges from 0 to 78, with a cutoff score of 20 or higher indicating possible disordered eating behaviors warranting further evaluation.

The EAT-26 has demonstrated strong psychometric properties across diverse populations. A reliability generalization meta-analysis involving 15 studies reported a pooled Cronbach’s alpha of 0.85 (95% CI: 0.81–0.88), suggesting high internal consistency (26). The analysis further indicated that the reliability of EAT-26 scores was not significantly influenced by sample characteristics such as language, age, or gender. In the present study, participants had the option to complete the survey in either Arabic or English, using previously validated versions of the scale appropriate for each language.

The final section included items assessing participants’ history of eating disorder diagnosis and treatment, as well as their perceived body image.

Ethical considerations

Ethical approval was obtained from the Institutional Review Board of Amran University (approval No. 854.) Informed consent was obtained electronically before participation, and respondents were assured of full anonymity and confidentiality. To reduce recall bias, the survey included clear and specific questions about recent behaviors. Data were stored securely and accessible only to the research team.

Statistical analysis

Data were analyzed using IBM SPSS Statistics version 28.0. Descriptive statistics were calculated to summarize participant characteristics. Categorical variables were presented as frequencies and percentages, while continuous variables were expressed as means and standard deviations. Normality of continuous variables was tested using the Kolmogorov–Smirnov test. To assess differences between groups, Chi-square tests were used for categorical variables. A binary outcome was generated based on the EAT-26 score, classifying students into two groups: those with disordered eating symptoms (score ≥ 20) and those without (score < 20). Multivariate

logistic regression analysis was conducted to identify independent predictors of disordered eating, including variables with a p-value < 0.20 in the bivariate analysis. A significance level of $p < 0.05$ was used to reject the null hypothesis.

Results

Prevalence of eating disorders

Out of the total 3540 participants, 30.4% (n = 1076) were identified as being at risk of disordered eating behaviors, based on EAT-26 scores of 20 or higher, while 69.6% (n = 2464) scored below this threshold, indicating no disordered eating behavior (Table 1). As illustrated in Figure 1, 31.6% of participants self-reported a prior diagnosis of an eating disorder, whereas 68.4% reported no such history.

Recent weight change

When asked about weight changes within the past six months, 23.7% (n = 838) of participants reported experiencing weight change, while the majority (76.3%, n = 2702) did not (Figure 2).

Sociodemographic and lifestyle correlates

A series of chi-square and Fisher's exact tests were conducted to explore the associations between disordered eating behaviors and various participant characteristics and the likelihood of disordered eating behaviors, presented in Table 1. No statistically significant differences were observed in relation to gender, smoking status, or qat chewing. However, other factors demonstrated associations with disordered eating risk. Participants among first-year students and sixth-year students were significantly more likely to exhibit disordered eating behaviors ($p < 0.001$). Marital status was also significantly associated with disordered eating ($p < .001$). Divorced and widowed individuals showed higher proportions of disordered eating compared to married and single participants.

Participants who engaged in regular physical activity were significantly less likely to exhibit disordered eating behaviors ($p < 0.001$). Conversely, individuals with a history of Type 1 Diabetes Mellitus (T1DM), gastrointestinal disease, autism, or sleeping disorders were notably more likely to be at risk ($p < 0.001$). Concern of psychological comorbidities, all examined psychological disorders except obsession were significantly associated with disordered eating (p

< 0.05). Additional risk factors included food insecurity, previous suicide attempts, and dissatisfaction with body weight, each showing significant associations with disordered eating (p < 0.001). Furthermore, parental perception of the participant being overweight was a particularly strong predictor, with 50.0% of those under such perceptions exhibiting behaviors indicative of disordered eating (p < 0.001).

Table 1. Sociodemographic, lifestyle, and health-related characteristics of participants, and their associations with disordered eating behavior

**Variable**	**Category**	**Total** 3540 N (column %)	**No Disordered Eating** (EAT-26 < 20) 2464 (69.6%) N (row %)	**At Risk of Disordered Eating** (EAT-26 ≥ 20) 1076 (30.4%) N (row %)	**p-value**
Gender[4]	Male	1644 (46.44%)	1160 (32.77%)	484 (13.67%)	0.256
	Female	1896 (53.56%)	1304 (36.84%)	592 (16.72%)	
Academic year[b]	First Year	611 (17.26%)	361 (10.2%)	250 (7.06%)	<0.001**
	Second Year	578 (16.33%)	384 (10.85%)	194 (5.48%)	
	Third Year	589 (16.64%)	407 (11.5%)	182 (5.14%)	
	Fourth Year	614 (17.34%)	465 (13.14%)	149 (4.2%)	
	Fifth Year	588 (16.61%)	440 (12.43%)	148 (4.18%)	
	Sixth Year	560 (15.82%)	407 (11.5%)	153 (4.32%)	
Marital status[b]	Married	546 (15.42%)	366 (10.34%)	180 (5.08%)	<0.001**
	Single	2818 (79.6%)	2017 (56.98%)	801 (22.63%)	
	Divorced	125 (3.53%)	59 (1.67%)	66 (1.86%)	
	Widowed	51 (1.44%)	22 (0.62%)	29 (0.82%)	

Smoking[b]	Yes	661 (18.67%)	441 (12.46%)	220 (6.21%)	0.074
	No	2879 (81.33%)	2023 (57.15%)	856 (24.18%)	
Qat chewing[a]	Yes	1413 (39.92%)	1002 (28.31%)	411 (11.61%)	0.179
	No	2127 (60.08%)	1462 (41.29%)	665 (18.79%)	
Regular sports practice[b]	Yes	982 (27.74 %)	623 (17.60%)	359 (10.14%)	<0.001**
	No	2558 (72.26%)	1841 (52.01%)	717 (20.25%)	
T1DM history[b]	Yes	302 (8.53%)	126 (3.56%)	176 (4.97%)	<0.001**
	No	3238 (91.47%)	2338 (66.05%)	900 (25.42%)	
GI disease history[b]	Yes	926 (26.16%)	564 (15.93%)	362 (10.23%)	<0.001**
	No	2614 (73.84%)	1900 (53.67%)	714 (20.17%)	
Autism history[b]	Yes	293 (8.28%)	119 (3.36%)	174 (4.92%)	<0.001**
	No	3247 (91.72%)	2345 (66.24%)	902 (25.48%)	
Sleeping disorders history[b]	Yes	859 (24.27%)	499 (14.10%)	360 (10.17%)	< 0.001*
	No	2681 (75.73%)	1965 (55.50%)	716 (20.23%)	
Parental view overweight[b]	Yes	740 (20.9%)	370 (10.45%)	370 (10.45%)	< 0.001*
	No	2800 (79.1%)	2094 (59.15%)	706 (19.95%)	
Parental pressure to eat[a]	Yes	1803 (50.93%)	1249 (35.28%)	554 (15.65%)	0.688
	No	1737 (49.07%)	1215 (34.32%)	522 (14.75%)	
Food insecurity[b]	Yes	938 (26.5%)	526 (14.86%)	412 (11.64%)	<0.001**
	No	2602 (73.5%)	1938 (54.75%)	664 (18.75%)	
	Yes	388 (11.0%)	152 (4.30%)	236 (6.70%)	<0.001**

Past Suicide Attempts[b]	No	3152 (89.0%)	2312 (65.30%)	840 (23.70%)	
Weight Satisfaction[b]	Satisfied	1904 (53.79%)	1377 (38.90%)	527 (14.89%)	<0.001**
	Dissatisfied	1636 (46.21%)	1087 (30.71%)	549 (15.50%)	
Psychological Comorbidities					
Post-traumatic stress disorder (PTSD)[b]	Yes	164 (4.63%)	75 (2.12%)	89 (2.51%)	<.001**
	No	3376 (95.37%)	2389 (67.49%)	987 (27.88%)	
Obsessive compulsive disorder (OCD)[b]	Yes	192 (5.42%)	100 (2.82%)	92 (2.60%)	<.001**
	No	3348 (94.58%)	2364 (66.78%)	984 (27.80%)	
Social anxiety disorder[b]	Yes	307 (8.67%)	176 (4.97%)	131 (3.70%)	<.001**
	No	3233 (91.33%)	2288(64.63%)	945 (26.69%)	
Bipolar disorder[a]	Yes	55 (1.55%)	16 (0.45%)	39 (1.10%)	<.001**
	No	3485 (98.45%)	2448 (69.15%)	1037 (29.30%)	
Schizophrenia[a]	Yes	42 (1.19%)	10 (0.28%)	32 (1.0%)	<.001**
	No	3498 (98.81%)	2454 (69.32%)	1044 (29.49%)	
Major depressive disorder[b]	Yes	112 (3.16%)	54 (1.53%)	58 (1.63%)	<.001**
	No	3428 (96.84%)	2410 (68.08%)	1018 (28.76%)	
Borderline personality disorder[b]	Yes	29 (0.82%)	14 (0.4%)	15 (0.42%)	<.001**
	No	3511 (99.18%)	2450 (69.21%)	1061 (29.97%)	
Perfectionism[b]	Yes	70 (2.0%)	38 (1.1%)	32 (0.90%)	.005*

	No	3470 (98.0%)	2426 (68.51%)	1044 (29.49%)	
Obsession[a]	Yes	60 (1.69%)	43 (1.21%)	17 (0.48%)	.726
	No	3480 (98.31%)	2421 (68.39%)	1059 (29.92%)	
Impulsiveness[b]	Yes	60 (1.69%)	34 (0.96%)	26 (0.73%)	.028*
	No	3480 (98.31%)	2430 (68.65%)	1050 (29.66%)	

a: Fisher's Exact Test; b: Chi-square Test; *p <.05; **p <.001.

Types and frequency of eating disorder symptoms

Among the 1117 participants who endorsed disordered eating symptoms, anorexia nervosa (8.6%), binge eating disorder (4.4%), and avoidant/restrictive food intake disorder (4.0%) were the most commonly reported diagnoses (Table 2). Symptoms varied in frequency, with 5.5% experiencing daily symptoms and 7.6% weekly. Regarding severity, 14.0% reported mild, 13.4% moderate, and 4.1% reported severe or very severe symptoms.

Table 2. Eating disorders symptoms and severity (N= 1117)

Variable	Frequency (n)	Percentage (%)
**Type of Eating disorders**		
Anorexia nervosa	303	8.6%
Bulimia nervosa	86	2.4%
Binge Eating disorder	157	4.4%
Avoidant/restrictive intake disorder	140	4.0%
Other	431	12.2%
**Frequency of Eating disorders symptoms**		

Daily	194	5.5%
Weekly	268	7.6%
Monthly	232	6.6%
Yearly	83	2.3%
Rarely	227	6.4%
Never	113	3.2%
**Severity of Eating disorders symptoms**		
Mild	496	14.0%
Moderate	476	13.4%
Severe	118	3.3%
Very severe	27	0.8%

Behavioral attitudes related to disordered eating

Table 3 outlines the prevalence of specific disordered eating behaviors among the participants. A substantial proportion, 40.1%, reported experiencing binge eating episodes at least once in their lifetime, with 13.8% engaging in such episodes on a weekly basis or more frequently. Induced vomiting as a method of weight control was reported by 27.5% of participants, with 10.3% indicating they engaged in this behavior at least once a month. Similarly, 27.6% of participants reported the use of laxatives, diet pills, or diuretics, with 7.8% using these substances on a weekly or more frequent basis. Excessive exercise, defined as engaging in physical activity for more than 60 minutes daily for the purpose of weight control, was reported by 3.7% of participants on a daily basis and by 4.1% several times a week.

Table 3. Frequency of behavioral attitudes related to Disordered Eating

Behavioral attitude questions	Never	Once a Month or Less	2–3 Times a Month	Once a Week	2–6 Times a Week	Once a Day or More
Gone on eating binges where you feel that you may not be able to stop?	2219 (59.9%)	633 (17.1%)	343 (9.3%)	261 (7.0%)	106 (2.9%)	145 (3.9%)
Ever made yourself sick (vomited) to control your weight or shape?	2650 (72.5%)	410 (11.2%)	266 (7.3%)	178 (4.9%)	65 (1.8%)	87 (2.4%)
Ever used laxatives, diet pills, or diuretics (water pills) to control your weight or shape?	2653 (72.4%)	371 (10.1%)	274 (7.5%)	188 (5.1%)	77 (2.1%)	100 (2.7%)
Exercise more than 60 minutes a day to lose or control your weight?	2220 (60.3%)	536 (14.6%)	384 (10.4%)	253 (6.9%)	149 (4.1%)	137 (3.7%)

Predictors of disordered eating behaviors

A binary logistic regression analysis (Table 4) was conducted to identify independent predictors of disordered eating behaviors. The history of GI disease, autism, and sleeping disorders did not independently predict disordered eating. However, engaging in regular sports practice was associated with a 24% increase in the odds of disordered eating. Having a history of T1DM also emerged as a significant predictor, increasing the odds by 40%.

Conversely, participants whose parents viewed them as overweight were over twice as likely to engage in disordered eating behaviors. Similarly, experiencing food insecurity increased the odds by approximately 43%, while a history of suicide attempts nearly doubled the odds. Notably, weight satisfaction was protective; participants satisfied with their weight were 16% less likely to engage in disordered eating.

Table 4. Multivariate logistic regression identifying significant predictors of Disordered Eating Behaviors

Predictors	OR	[95% CI]	p-value
Regular sport practice	1.24	[1.039, 1.471]	.017*
T1DM History	1.40	[1.044, 1.886]	.025*
GI Disease History	1.06	[.887, 1.271]	.516
Autism History	1.33	[.987, 1.795]	.061
Sleeping Disorders History	1.12	[.929, 1.350]	.233
Parental View Overweight	2.02	[1.679, 2.438]	<.001**
Food Insecurity	1.43	[1.198, 1.704]	<.001**
Suicide Attempts	1.94	[1.500, 2.515]	<.001**
Weight satisfaction	.84	[.714, .978]	.025*
Post-traumatic stress disorder	2.20	[1.541, 3.135]	<.001**
Obsessive compulsive disorder	2.22	[1.613, 3.066]	<.001**
Social anxiety disorder	2.11	[1.627, 2.725]	<.001**
Bipolar disorder	3.52	[1.856, 6.677]	<.001**
Schizophrenia	5.96	[2.805, 12.677]	<.001**
Major depressive disorder	2.66	[1.767, 3.996]	<.001**
Borderline personality disorder	1.98	[.910, 4.299]	.085
Perfectionism	2.01	[1.210, 3.352]	.007*
Impulsiveness	2.65	[1.546, 4.542]	<.001**

Note. OR = Odds Ratio; CI = Confidence Interval; *$p < .05$; **$p < .001$.

Several psychological comorbidities were significant predictors. PTSD, OCD, social anxiety disorder, major depressive disorder, perfectionism, and impulsiveness was associated with more than double the odds of disordered eating. Notably, bipolar disorder (OR = 3.52, $p < .001$) and schizophrenia (OR = 5.96, $p < .001$) were associated with the highest increases in odds. While borderline personality disorder did not reach conventional levels of statistical significance ($p = .085$).

Discussion

Our cross-sectional study (N=3540) revealed a high prevalence of disordered eating risk among medical trainees, with 30.4% screening positive (EAT-26 ≥20). Disordered eating was significantly associated with several factors such as regular sports practice, histories of type 1 diabetes mellitus (DM), weight dissatisfaction, food insecurity, past suicide attempts, parental perception of being overweight, and the presence of psychological comorbidities (PTSD, OCD, bipolar disorder, and schizophrenia). Furthermore, multiple concerning behaviors were reported. Those comprise binge eating (40.1% ever), self-induced vomiting (27.5% ever), laxative/diet pill misuse (27.6% ever), excessive exercise for weight control (39.7% ever), and significant recent weight loss (23.7%). Other factors, such as marital status, smoking, Qat chewing, and parental pressure to eat, showed no significant association with the risk of disordered eating.

Our finding that 30.4% of medical trainees screened positive for disordered eating exceeds the global pooled prevalence estimates of 17.35% by Fekih-Romdhane et al. (2022) and 15.1% by Jahrami et al. (2024) for medical students (27,28). This higher rate aligns with recent data from the MENA region, where Belhaj Salem et al. (2025) reported 24.8% at risk, with specific countries like Egypt, Saudi Arabia, and the UAE reporting rates of 32-35% (29). Whereas our female participants exhibited a higher risk (16.7%) than males (13.7%), this difference was not statistically significant ($p=.256$). Such a lack of significant difference is contrary to the common belief that females are more commonly affected by eating disorders. Nonetheless, the meta-analysis by Fekih-Romdhane et al. (2022) reported no difference between genders among medical students (28). In addition, this observation is supported by recent literature emphasizing the significant proportion of males affected by eating disorders (30) Our study reinforces that

medical trainees consistently exhibit higher disordered eating prevalence than the general population (10.4% vs. 5.7%) in females, particularly within non-Western cultures (28,31).

Medical students exhibit heightened vulnerability to eating disorders (EDs) due to interlocking academic, psychological, and sociocultural risk factors. The inherently demanding training environment, characterized by heavy workloads, intense competition, high expectations, and exposure to illness and mortality, creates significant chronic stress (27,32,33). This stress co-occurs with or precipitates high rates of depression, anxiety, burnout, and perfectionism, which our study strongly linked to ED risk (27,32,33) .Maladaptive coping mechanisms, such as disordered eating behaviors, may emerge when effective stress outlets are lacking (27,31,33). Moreover, the pervasive internalization of the "thin ideal" body type promoted in media and sometimes perceived as linked to professionalism, adds to body dissatisfaction (27,33). Critical attitudes towards weight and parental perception of the student as overweight were also reported as significant predictors of disordered eating in previous studies (31,33). Notably, recent intervention work shows that directly addressing weight self-stigma and body-image concerns can meaningfully improve outcomes (Al Maghaireh, Basyouni, Alsaqer, et al., 2024).

We demonstrated a significant relationship between EDs and DM but not GI disorders. Students with a history of type 1 DM were significantly more likely to screen positive for disordered eating. DM management necessitates intense focus on food, weight, and insulin dosing, which can foster detrimental attitudes and behaviors like diabulimia, deliberate insulin restriction for weight control, affecting 20-60% of individuals with type 1 DM and carrying severe complications and a 3.2-fold increased mortality risk (34–36). Conversely, ED behaviors (binge eating, purging, restriction) disrupt glycemic control and can lead to diabetes development [8, (34,36). Likewise, GI disorders and EDs have a significant overlap. Our observed 26.2% GI history aligns with the high prevalence of disordered eating (up to 44%) in GI populations (37). GI symptoms are a primary driver for Avoidant/Restrictive Food Intake Disorder (ARFID), characterized by food avoidance due to fear of consequences like pain or vomiting, not body image concerns (38,39) Going the other direction, EDs cause severe GI sequelae, including dental erosion, esophageal injury, gastroparesis, constipation, and IBS-like symptoms (40). This discombobulating interplay, exacerbated by shared comorbidities like anxiety and depression,

creates diagnostic challenges and explains why it became non-significant after accounting for the presence of other psychiatric comorbidities (28,37,39,41)

Psychiatric conditions and suicidality were significantly associated with EDs in our study, with 11.0% of participants reporting attempts. This is consistent with established evidence that suicidality remains a grave concern in EDs (42,43) Anorexia Nervosa (AN) carries an alarmingly high suicide mortality risk, up to 200 times greater than the general population (43)]. Lifetime suicide attempt rates range from 3-20% in AN to 25-35% in Bulimia Nervosa (BN) (42). Moreover, psychiatric comorbidity is the norm in Eds and is present in up to 96% of clinical samples (44) Our findings confirm strong associations between ED risk and self-reported psychological comorbidities, such as social anxiety (3.7%), OCD (2.6%), PTSD (2.5% of at-risk group), bipolar disorder (1.1%), and schizophrenia (0.9%). Several previous studies link mood disorders like Major Depressive Disorder and anxiety disorders to ED in different populations (41,44). Furthermore, trauma and PTSD were suggested to be the major drivers of ED complexity and worse outcomes (44,45).

Our study entertains a large sample size and spans all academic years in medical school and employs a validated screening tool (EAT-26), which increases the generalizability of our findings. However, our results have to be interpreted with several considerations. The causality between various factors and EDs cannot be determined due to the cross-sectional nature of our study. Similarly, we relied on self-reported data, which may have introduced some recall bias. Prospective cohorts should track medical trainees from enrollment through residency. This will clarify causal pathways between academic stress, comorbidities, and ED onset. Moreover, institutions are advised to establish collaborative care models bridging ED services with depression, anxiety, and suicide prevention programs, and prioritize students with reported comorbidities.

Conclusion

This study found a concerning prevalence of disordered eating (30.4%) among medical trainees, significantly higher than global estimates. Risk factors included psychological comorbidities, diabetes, weight dissatisfaction, and suicidality, emphasizing the interplay of medical, psychological, and sociocultural influences. The demanding medical training environment likely contributes to this vulnerability. Our findings also challenge gender stereotypes by revealing

comparable risk between males and females. The bidirectional relationships between eating disorders, chronic diseases, and mental health conditions necessitate integrated care approaches. Despite limitations in causal inference due to the cross-sectional design, these results call for urgent institutional action, including routine screening, targeted interventions, and longitudinal research to better understand and mitigate eating disorder risk in this critical population. Protecting future healthcare providers requires prioritizing their mental and physical well-being throughout training.

Acknowledgments

We would like to express our deepest gratitude to the Yemeni medical student community for their invaluable support and active participation in this research. Their collaboration, commitment, and enthusiasm toward advancing medical knowledge were fundamental to the success of this study.

Our heartfelt thanks go to Professor Mohammed Abdullah Al-Eryani for his generous support and steadfast encouragement throughout all stages of this research. His continuous guidance and belief in the potential of this work played a vital role in its completion and quality.

We also extend our sincere appreciation to Professor Mohammed Alshehri for his exceptional mentorship. His insightful feedback and thoughtful direction significantly enhanced the academic rigor and impact of this study.

Special thanks are due to Saif Alaribi and Yusra Rashed Al-Subaihi, whose assistance during the data collection phase, though limited in scope, meaningfully contributed to the progress of this project.

We are also grateful to the following individuals for their dedicated efforts in data collection:

Mohammed R. Arrabyee, Ziad Mohammed AL-Othrubi, Amjed Al-Jahmi, Hajar Aqabi, Akram Al-Wadei, Fatima Odhah, Khulood Al-Khateeb, Fatima Al-Mutawakel, Ahmed Ahmed, Yousef Al-Qadimi, and Akram Al-Wadei.

Their valuable contributions were instrumental in ensuring the thoroughness and success of the study.

Author Contributions

Naji Al-bawah and Mohamed Baklola contributed equally to the conception, study design, data analysis, and manuscript drafting.

Anas H. Khalifeh contributed to the methodological framework, literature review, and critical revision of the manuscript.

Ahmed Al-Eryani assisted in data interpretation, statistical verification, and manuscript editing.

Anas Zakarya Nourelden participated in survey development and supervised data collection.

Saif Alaribi was involved in participant recruitment and initial data gathering.

Ehab Sharyan supported the coordination of field data collection and quality assurance.

Fayda Al-Wesabi and Aaida Al-Wesabi contributed to transcription, data cleaning, and entry.

Ahdab Al-Jaberi and Sama Shamsan provided support in follow-up communications and data validation.

Ahmed Abdulmughni contributed to visualization and formatting of study results.

Ghailan Al-Jarbani assisted in literature sourcing and background synthesis.

Hisham Salman helped with ethical approval processes and logistical support.

Amira Yasmine Benmelouka provided final proofreading, formatting for submission, and contributed to manuscript clarity.

Data Availability

The data that support the findings of this study are available from the corresponding author upon reasonable request.

Funding

This research received no specific grant from any funding agency in the public, commercial, or not-for-profit sectors.

Competing Interests

The authors declare that they have no competing interests.

References

1. Attia E, Becker AE, Bryant-Waugh R, Hoek HW, Kreipe RE, Marcus MD, et al. Feeding and eating disorders in DSM-5. American Journal of Psychiatry. 2013;170(11):1237–9.

2. Tavolacci MP, Grigioni S, Richard L, Meyrignac G, Déchelotte P, Ladner J. Eating disorders and associated health risks among university students. Journal of nutrition education and behavior. 2015;47(5):412–20.

3. Phillips L, Kemppainen JK, Mechling BM, MacKain S, Kim-Godwin Y, Leopard L. Eating disorders and spirituality in college students. Journal of Psychosocial Nursing and Mental Health Services. 2015;53(1):30–7.

4. Call C, Walsh BT, Attia E. From DSM-IV to DSM-5: changes to eating disorder diagnoses. Current opinion in psychiatry. 2013;26(6):532–6.

5. Ghazzawi HA, Nimer LS, Sweidan DH, Alhaj OA, Abulawi D, Amawi AT, et al. The global prevalence of screen-based disordered eating and associated risk factors among high school students: systematic review, meta-analysis, and meta-regression. Journal of Eating Disorders. 2023;11(1):128.

6. Solmi M, Monaco F, Højlund M, Monteleone AM, Trott M, Firth J, et al. Outcomes in people with eating disorders: A transdiagnostic and disorder-specific systematic review, meta-analysis and multivariable meta-regression analysis. World Psychiatry. 2024;23(1):124–38.

7. Galmiche M, Déchelotte P, Lambert G, Tavolacci MP. Prevalence of eating disorders over the 2000–2018 period: a systematic literature review. The American journal of clinical nutrition. 2019;109(5):1402–13.

8. Jahrami H, Sater M, Abdulla A, Faris MAI, AlAnsari A. Eating disorders risk among medical students: a global systematic review and meta-analysis. Eating and Weight Disorders-Studies on Anorexia, Bulimia and Obesity. 2019;24(3):397–410.

9. Jahrami H, Saif Z, Trabelsi K, Ghazzawi H, Pandi-Perumal SR, Seeman M V. An Umbrella Review and a Meta-analysis of Meta-analyses of Disordered Eating Among Medical Students. Alpha psychiatry. 2024 Mar;25(2):165–74.

10. Zeng W, Chen R, Wang X, Zhang Q, Deng W. Prevalence of mental health problems among medical students in China: A meta-analysis. Medicine. 2019 May;98(18):e15337.

11. Dodin Y, Obeidat N, Dodein R, Seetan K, Alajjawe S, Awwad M, et al. Mental health and lifestyle-related behaviors in medical students in a Jordanian University, and variations by clerkship status. BMC medical education. 2024 Nov;24(1):1283.

12. Adhikari A, Dutta A, Sapkota S, Chapagain A, Aryal A, Pradhan A. Prevalence of poor mental health among medical students in Nepal: a cross-sectional study. BMC medical education. 2017 Nov;17(1):232.

13. Hammad NM, Sonneville KR, Shanab BM, Khosla P, Austin SB, Leung CW. Weight bias internalization, weight controllability beliefs, and probable eating disorder associations among medical students at a private university in the US. Eating behaviors. 2025 Jul;58:102010.

14. Mallaram GK, Sharma P, Kattula D, Singh S, Pavuluru P. Body image perception, eating disorder behavior, self-esteem and quality of life: A cross-sectional study among female medical students. Journal of eating disorders. 2023;11(1):225.

15. Abaatyo J, Rukundo GZ, Twine M, Lutasingwa D, Favina A, Nyemara N, et al. Psychological distress among healthcare professionals in Mbarara, following the 2022 Ebola Virus Disease outbreak in Uganda: a mixed methods study. BMC psychiatry. 2024;24(1):469.

16. Puthran R, Zhang MWB, Tam WW, Ho RC. Prevalence of depression amongst medical students: A meta-analysis. Medical education. 2016;50(4):456–68.

17. Abdulla ZARA, Almahmood HO, Alghasra RR, Alherz ZAS, Alsharifa HAG, Qamber SJ, et al. Prevalence and associated factors of binge eating disorder among Bahraini youth and young adults: A cross-sectional study in a self-selected convenience sample. Journal of Eating Disorders. 2023;11(1):5.

18. Elsherif ES, Al-Rakhawy MY, Fathy H, Adel A, Khalaf OO. Disordered eating behaviors among a sample of final-year medical students at Cairo University and its relationship with alexithymia and social cognition. Middle East Current Psychiatry. 2025;32(1):4.

19. Ali EAA, Shehata WM. Eating disorder risk among medical students at Tanta University, Egypt. Egyptian J Community Med. 2020;38(4):17.

20. Ogundele MO. Behavioural and emotional disorders in childhood: A brief overview for paediatricians. World journal of clinical pediatrics. 2018;7(1):9.

21. Golden NH. Eating disorders in adolescence and their sequelae. Best Practice & Research Clinical Obstetrics & Gynaecology. 2003;17(1):57–73.

22. Almahmeed MB, Almutawa MA, Naguib YM. The prevalence of and the effect of global stressors on eating disorders among medical students. Frontiers in psychology. 2025;16:1507910.

23. Alhaj OA, Fekih-Romdhane F, Sweidan DH, Saif Z, Khudhair MF, Ghazzawi H, et al. The prevalence and risk factors of screen-based disordered eating among university students: a

global systematic review, meta-analysis, and meta-regression. Eating and Weight Disorders - Studies on Anorexia, Bulimia and Obesity. 2022;27(8):3215–43.

24. Belhaj Salem S, Idrissi FE El, Fikry Mohamed A, Khrais H, Jad RE, Haq UU, et al. Screening of the Risk of Eating Disorders Among Medical Students in the MENA region and Its Associated Factors: A Multinational Cross-Sectional Study. Brain and behavior. 2025 Mar;15(3):e70417.

25. Garner DM, Olmsted MP, Bohr Y, Garfinkel PE. The eating attitudes test: psychometric features and clinical correlates. Psychological medicine. 1982;12(4):871–8.

26. Janahi S, Alkhater N, Bucheer A, Hashem Y, Alothman KK, Alsada A, et al. A Reliability Generalization Meta-Analysis of the Eating Attitudes Test 26 (EAT-26) Scale. Cureus. 2024 Nov;16(11):e73647.

27. Jahrami H, Saif Z, Trabelsi K, Ghazzawi H, Pandi-Perumal SR, Seeman M V. An Umbrella Review and a Meta-analysis of Metaanalyses of Disordered Eating Among Medical Students. ALPHA PSYCHIATRY. 2024 May 2;25(2):165–74.

28. Fekih-Romdhane F, Daher-Nashif S, Alhuwailah AH, Al Gahtani HMS, Hubail SA, Shuwiekh HAM, et al. The prevalence of feeding and eating disorders symptomology in medical students: an updated systematic review, meta-analysis, and meta-regression. Eating and Weight Disorders - Studies on Anorexia, Bulimia and Obesity. 2022 Jan 24;27(6):1991–2010.

29. Belhaj Salem S, Idrissi FE El, Fikry mohamed A, Khrais H, Jad RE, Haq UU, et al. Screening of the Risk of Eating Disorders Among Medical Students in the MENA region and Its Associated Factors: A Multinational Cross-Sectional Study. Brain Behav. 2025 Mar 18;15(3).

30. Gorrell S, Murray SB. Eating Disorders in Males. Child Adolesc Psychiatr Clin N Am. 2019 Oct;28(4):641–51.

31. Almahmeed MB, Almutawa MA, Naguib YM. The prevalence of and the effect of global stressors on eating disorders among medical students. Front Psychol. 2025 Feb 4;16.

32. Mallaram GK, Sharma P, Kattula D, Singh S, Pavuluru P. Body image perception, eating disorder behavior, self-esteem and quality of life: a cross-sectional study among female medical students. J Eat Disord. 2023 Dec 15;11(1):225.

33. Motorga R, Ionescu M, Nechita F, Micu D, Băluțoiu I, Dinu MM, et al. Eating disorders in medical students: prevalence, risk factors, comparison with the general population. Front Psychol. 2025 Jan 7;15.

34. Atriham AR, Kleszczyński J, Sierakowska A, Springer J. Diabulimia – a diagnostic and therapeutic challenge in the emergency department. Eur J Transl Clin Med. 2024 Mar 22;7(1):57–62.

35. Coleman SE, Caswell N. Diabetes and eating disorders: an exploration of 'Diabulimia.' BMC Psychol. 2020 Dec 23;8(1):101.

36. Dziewa M, Bańka B, Herbet M, Piątkowska-Chmiel I. Eating Disorders and Diabetes: Facing the Dual Challenge. Nutrients. 2023 Sep 12;15(18):3955.

37. Soliman OM, Mikocka-Walus A, Warner MM, Skvarc D, Olive L, Knowles SR. Systematic Review and Meta-Analysis: Examining the Psychometric Evaluations of Disordered Eating Scales in Adults Living With Gastrointestinal Conditions. Neurogastroenterology & Motility. 2025 May 10;37(5).

38. Brosig L, Düplois D, Hiemisch A, Kiess W, Hilbert A, Schlensog-Schuster F, et al. Birth-related, medical, and diagnostic characteristics in younger versus older children with avoidant/restrictive food intake disorder (ARFID). J Eat Disord. 2023 Oct 26;11(1):190.

39. Che Y, Li S, Hu X, Hu H, Zhang C, He C. Avoidant/Restrictive Food Intake Disorder in Adult Patients with Gastrointestinal Diseases: A Concept Analysis Using Evolutionary Approach. Risk Manag Healthc Policy. 2025 Jun;Volume 18:2063–80.

40. Lin JA, Woods ER, Bern EM. Common and Emergent Oral and Gastrointestinal Manifestations of Eating Disorders. Vol. 17, Gastroenterology & Hepatology. 2021.

41. Garcia SC, Mikhail ME, Keel PK, Burt SA, Neale MC, Boker S, et al. Increased rates of eating disorders and their symptoms in women with major depressive disorder and anxiety disorders. International Journal of Eating Disorders. 2020 Nov 26;53(11):1844–54.

42. Franko DL, Keel PK. Suicidality in eating disorders: Occurrence, correlates, and clinical implications. Clin Psychol Rev. 2006 Oct;26(6):769–82.

43. Pisetsky EM, Thornton LM, Lichtenstein P, Pedersen NL, Bulik CM. Suicide attempts in women with eating disorders. J Abnorm Psychol. 2013 Nov;122(4):1042–56.

44. Brewerton TD. The integrated treatment of eating disorders, posttraumatic stress disorder, and psychiatric comorbidity: a commentary on the evolution of principles and guidelines. Front Psychiatry. 2023 May 12;14.

45. Vanzhula IA, Calebs B, Fewell L, Levinson CA. Illness pathways between eating disorder and post-traumatic stress disorder symptoms: Understanding comorbidity with network analysis. European Eating Disorders Review. 2019 Mar 7;27(2):147–60.

 Click here to access/download;Figure;Figures.docx

Figure 1. Self-reported history of Eating disorder diagnosis among participants

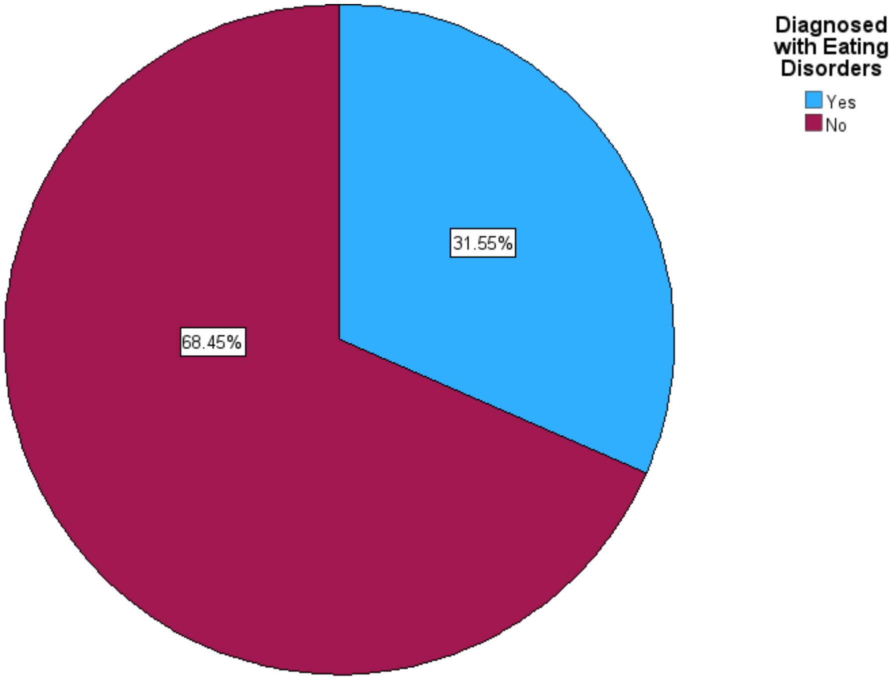

Figure 2. Self-reported weight change among participants in the past six months

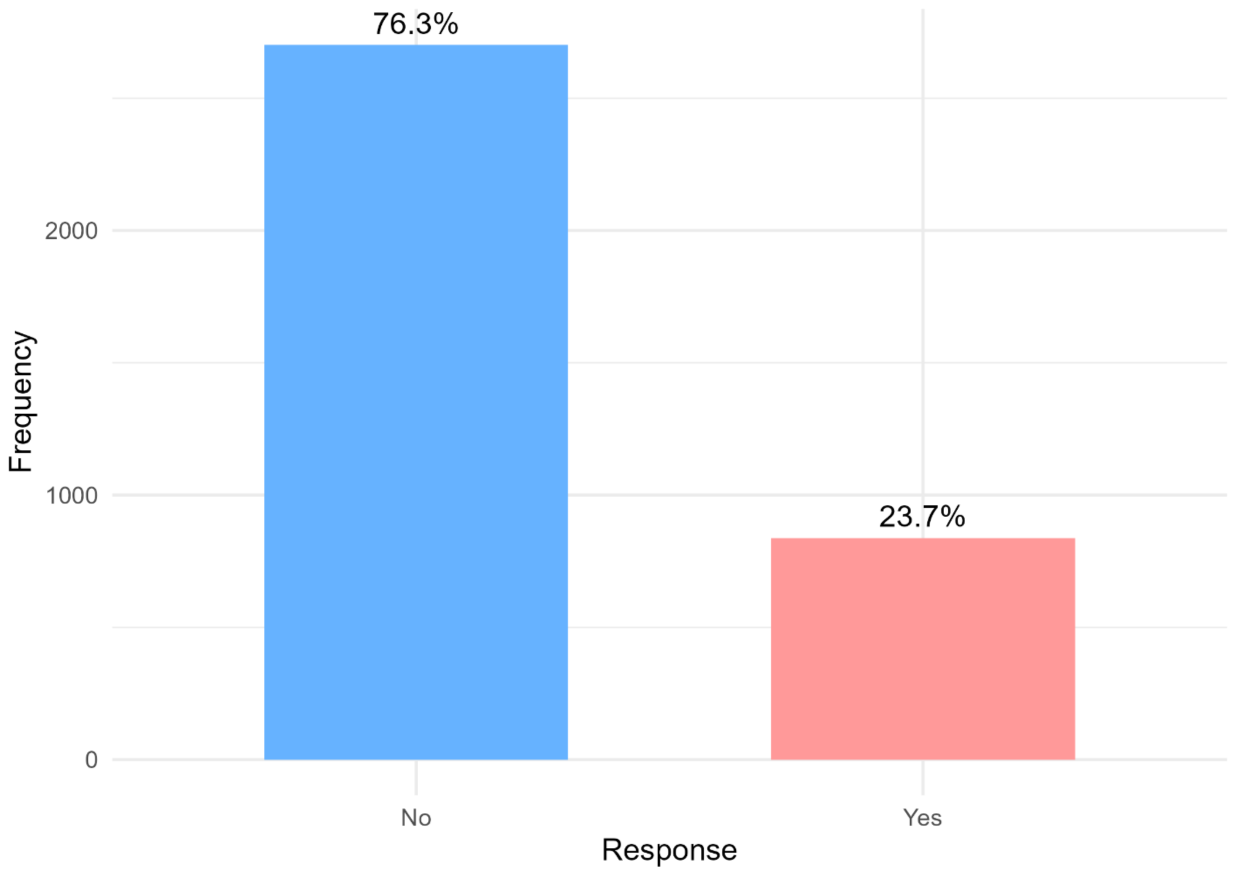

Click here to access/download
Supporting Information
ethical a.pdf
