# Supplementary material for: Prevalence and risk factors of disordered eating risk among Yemeni medical students: A national multicenter cross-sectional study
Source: PLoS One. 2026 Mar 25;21(3):e0345771. doi: 10.1371/journal.pone.0345771 (PMC13016294; doi:10.1371/journal.pone.0345771)
Supplement: Attachment — Submitted filename: Point to point response.pdf [file pone.0345771.s005.pdf]

Response to reviewers

We sincerely thank the Editor and the Reviewers for their careful evaluation of our manuscript and for their constructive and insightful comments. We have revised the manuscript thoroughly to address all concerns raised. Below, we provide a detailed, point-by-point response. All changes have been incorporated into the revised manuscript.

Reviewer 1

Comment 1: *Did you conduct a pilot study?*

Response: Yes, a pilot study was conducted prior to the main data collection. We have now explicitly reported this in the Methods section. The questionnaire was pilot tested among 40 medical students who were not included in the final analysis to assess clarity, cultural appropriateness, and technical functionality of the online survey. Minor linguistic and formatting modifications were made based on participant feedback. We also reported the internal consistency of the EAT-26 in the pilot sample using Cronbach alpha.

Comment 2: *What are the practical implication of your study*

Response: Thank you for this important suggestion. We have expanded the Discussion section to clearly outline the practical implications of our findings. Specifically, we now emphasize the need for routine screening for disordered eating risk within medical schools, integration of mental health and nutritional support services, and targeted interventions for students with identified psychological and social risk factors. These implications are discussed in the revised Discussion and Conclusion sections.

Comment 3: *Language and gramaticalr revision are needed*

Response: We appreciate this comment. The manuscript has undergone comprehensive language and grammatical revision. Sentence structure, clarity, academic tone, and terminology consistency were carefully improved throughout the manuscript. Redundant phrasing was

removed, and terminology was standardized to reflect screening for disordered eating risk rather than diagnosed eating disorders.

Reviewer 2

We thank Reviewer 2 for their detailed and constructive feedback, which greatly improved the clarity, rigor, and scientific precision of the manuscript.

Title

Comment 1: *The title requires revision for scientific precision. The term (Uncovering) is stylistic rather than scientific and implies discovery or novelty, which is not necessary here.*

Response: We agree with this comment and have revised the title to adopt a more neutral and scientific tone. The revised title removes stylistic language and accurately reflects the study design and outcomes.

Comment 2: *The title refers to (Eating Disorders), which may imply clinically diagnosed conditions. However, the study assesses risk of disordered eating using the EAT-26.*

Response: This is an important point. The title has been revised to replace “Eating Disorders” with “Disordered Eating Risk,” ensuring consistency with the screening nature of the EAT-26 and avoiding any implication of clinical diagnosis.

Abstract

Comment 3: *The Results section does not clearly report sociodemographic, lifestyle, and health-related characteristics.*

Response: The Abstract has been revised to clearly summarize key sociodemographic, medical, psychological, and social correlates of disordered eating risk identified in the study.

Comment 4: *Protective factors are reported in the Results but are not reflected in the title or study objective.*

Response: We clarified the study objective to include both risk and protective factors. Protective factors are now consistently reported and interpreted in the Abstract, Results, and Discussion sections.

Comment 5: *Terminology is inconsistent between the title and conclusion.*

Response: Terminology has been standardized throughout the manuscript. The terms "disordered eating risk" and "screening positive on the EAT-26" are now used consistently from the Abstract through the Conclusion.

Methods

Comment 6: *The manuscript uses multiple terms interchangeably despite using a screening tool.*

Response: We fully agree. All terminology has been standardized to reflect screening outcomes. References to "eating disorders" are now limited to background context, while study findings consistently refer to "disordered eating risk" or "disordered eating behaviors."

Comment 7: *Clarify the method used to select institutions and participants and provide geographic details.*

Response: We have clarified the selection of participating medical colleges, including the number of institutions, their geographic distribution across multiple Yemeni governorates, and the recruitment approach. Terminology has been unified to "medical colleges" throughout the manuscript.

Comment 8: *Sample size calculation using G*Power requires justification.*

Response: We have expanded the Sample Size Calculation subsection to justify the use of G*Power software, explain parameter selection, and cite its widespread use in epidemiological and regression-based studies.

Comment 9: *Inclusion and exclusion criteria are repetitive, and exclusion criteria are missing.*

Response: This section was streamlined. Exclusion criteria were clearly added, repetition was removed, and the rationale for inclusion criteria was clarified. The process of voluntary participation and electronic informed consent was also explicitly described.

Comment 10: *Clarify questionnaire structure, validation, language versions, and permission for EAT-26 use.*

Response: The Methods section was revised to introduce the questionnaire clearly under a "Study Tools" subsection. We specified that the EAT-26 is a freely available screening tool, described the Arabic and English validated versions used, reported internal consistency using Cronbach alpha, and attached the questionnaire as supplementary material.

Comment 11: *Statistical analysis requires additional details regarding regression assumptions and variable selection.*

Response: We expanded the Statistical Analysis section to include the number of outcome events, justification for $p < 0.20$ variable selection, assessment of multicollinearity, model fit testing, and clarification of how variables were entered into the regression model.

Results

Comment 12: *The Results section should begin with a descriptive summary.*

Response: The Results section was restructured to begin with a concise description of participant characteristics and prevalence of disordered eating risk before presenting associations.

Comment 13: *Tables are overloaded, inconsistent, and unclear.*

Response: Table 1 was split into two separate tables to improve clarity. All tables were revised to ensure consistent terminology, correct percentage calculations, clear denominators, and alignment with the text. Tables were renumbered accordingly.

Discussion

Comment 14: *There are inconsistencies regarding marital status and prevalence comparisons.*

Response: We clarified that marital status was significant only in bivariate analysis and did not remain significant in multivariable regression. Prevalence comparisons were revised to explicitly acknowledge differences in study design and screening tools, and unsupported statements were removed or softened.

Conclusion

Comment 15: *The Conclusion should focus on key findings, with limitations addressed elsewhere.*

Response: The Conclusion was rewritten to focus strictly on the main findings and implications. Limitations were moved to a dedicated subsection within the Discussion, as recommended.

References

Comment 16: *Some references require updating.*

Response: All references were reviewed and updated where necessary to ensure accuracy and currency.
