# Supplementary material for: Prevalence and risk factors of disordered eating risk among Yemeni medical students: A national multicenter cross-sectional study
Source: PLoS One. 2026 Mar 25;21(3):e0345771. doi: 10.1371/journal.pone.0345771 (PMC13016294; doi:10.1371/journal.pone.0345771)
Supplement: Attachment — Submitted filename: Reviewer Comments_Final.docx [file pone.0345771.s002.docx]

**Reviewer Comments**

**Manuscript Title:** Uncovering the Burden and Risk Factors of Eating Disorders Among Yemeni Medical Students: A National Multicenter Cross-Sectional Analysis

**Title**

- The title requires revision for scientific precision. The term **(Uncovering)** is stylistic rather than scientific and implies discovery or novelty, which is not necessary here. Replacing it would improve neutrality and clarity.
- More importantly, the title refers to **(Eating Disorders),** which may imply clinically diagnosed conditions. However, the study assesses **risk of disordered eating using the EAT-26**, not diagnosed eating disorders.
- Please standardize terminology based on your outcome (EAT-26 ≥20) from the introduction through the conclusion. I recommend using "disordered eating risk" or "**risk of disordered eating behaviors"** consistently.

**Abstract**

- The Results section does not clearly report **sociodemographic, lifestyle, and health-related characteristics**, although these are mentioned.
- Protective factors are reported in the Results but are **not reflected in the title or study objective**; this inconsistency should be addressed.
- The Conclusion uses the terms **“disordered eating behaviors”** and **“at risk based on EAT-26,”** which aligns with the methods but conflicts with the title’s reference to “eating disorders.” Terminology should be consistent throughout.
- Please revise some sentences for clarity and professional flow.

**Introduction**

- The objective is generally reflected in the title through terms such as “burden” and “risk factors.” However, for accuracy, the title should explicitly indicate **risk of disordered eating** rather than diagnosed eating disorders.

**Methods**

**Terminology**

- The manuscript uses multiple terms interchangeably (**eating disorders, disordered eating, disordered eating behaviors**), despite using the **EAT-26**, which measures risk of disordered eating. Please standardize terminology throughout according to the measurement tool.

**Study Setting and Participants**

- Please clarify:
  - The **method used to select institutions and participants**.
  - The **names of universities and cities** included to support claims of broad geographical representation.
  - Whether all institutions were located in **Sana’a** or across different regions in Yemen.
- There is inconsistency between “universities in Yemen” (Abstract) and “institutions” (Methods). Please unify terminology.

**Sample Size**

- The use of **G*Power** for sample size calculation should be supported by:
  - Justification for selecting this method.
  - Relevant parameters and references.

**Inclusion and Exclusion Criteria**

- There is repetition in the presentation of inclusion criteria; please streamline.
- Exclusion criteria are not reported and should be clearly stated.
- The criterion “age ≥18 years” adds limited value, as undergraduate medical students are typically adults; please justify its inclusion.
- Clarify how **voluntary participation** and **electronic informed consent** were obtained and documented.

**Instruments**

- Before detailing sections, introduce the **overall questionnaire** used in the study. State clearly of this subsection that a structured, self-administered online questionnaire was used?
- Regarding the **EAT-26**:
  - Specify whether a free or licensed version was used and whether formal permission was obtained.
  - Please attach the questionnaire and EAT-26 tool you have used.
  - Provide details on **validation**, including reliability, internal consistency, and any face or content validation performed.
  - Clarify whether the **Arabic and/or English versions** were used.
  - The phrase (previously validated versions) requires clarification and **supporting references**.
  - Please discuss whether these validated versions are appropriate for **Yemeni medical students**.

**Study Design**

- Specify whether the study was conducted **electronically, face-to-face, or both**.
- Clarify whether the questionnaire was **self-administered**.

**Statistical Analysis**

- The overall statistical plan is appropriate; however, additional details are required to confirm rigor:
  - Report the **number of participants with EAT-26 ≥20** and whether the **events-per-variable** criterion was satisfied for logistic regression.
  - Indicate whether **multicollinearity** and **model fit diagnostics** were assessed.
  - Clarify whether continuous predictors were modeled as **continuous or categorical**, and whether linearity was assessed.
  - The use of the **Kolmogorov–Smirnov test** should be clarified. Since normality is not an assumption of logistic regression, please explain that it was used to guide **data presentation** (mean ± SD vs. median [IQR]).
  - Specify the **variable selection method** used in the final regression model (e.g., enter, stepwise).
  - Please justify the use of **p < 0.20** for variable selection in the multivariable logistic regression and provide the corresponding bivariate analysis results, as they are not included in the manuscript.

**Results**

- The Results section should begin with a **brief descriptive summary of participant demographics**.
- The **prevalence of disordered eating** is not clearly explained. Although referenced in **Table 1**, it does not appear to be calculated there—please clarify.
- Results consistently describe **“risk of disordered eating”**, not eating disorders, which conflicts with the title.
- Some variables in **Table 1** (e.g., **academic year, marital status**) appear inconsistently interpreted; please recheck and align text with tables.
- **Table 3:** The title and text should be consistent (behavioral attitudes vs. disordered eating behaviors). Percentages should be carefully reviewed.
- Please check and correct the **percentage totals** in your tables, as some appear to sum to 100.1% or 99.9%.
- **Table 4:**
  - Ensure consistency between the table title and the text description.
  - Clarify the rationale for reporting **two p-value thresholds** (*p* <0.05; **p* <0.001).
  - In the Methods, you state that students were classified into two groups based on EAT-26, but this classification is not clearly presented in the tables.
  - Add **abbreviations and footnotes** for clarity.
  - Explain why certain significant **sociodemographic predictors** were not included in the final model.

**Discussion**

- There is an inconsistency regarding **marital status**: it is reported as non-significant in the Discussion, yet Table 1 shows a significant association (*p* <0.001).
- The statement (Our study reinforces that medical trainees consistently exhibit higher disordered eating prevalence than the general population (10.4% vs. 5.7%) in females, particularly within non-Western cultures (28,31), is not clearly supported by the study’s own data; please clarify or remove.
- The statement (Our finding that 30.4% of medical trainees screened positive for disordered eating exceeds the global pooled prevalence estimates..), please specify whether the Prevalence calculation and screening tools used in these global studies are comparable to those used in the present study?

**Conclusion**

- The Conclusion should focus strictly on **key findings**. Limitations and strengths are better placed in a dedicated section or at the end of the Discussion.
- No need to mention and repeat the **percentage** in the conclusion.

**Acknowledgments**

- The Acknowledgments section is overly long and should be shortened.

**References**

- Some references require updating (e.g., references **21 and 25**).

**Based on the detailed comments in the provided PDF, please revise the manuscript to address all remaining issues identified therein.**

**Recommendation: Major Revision**

**I recommend the manuscript be revised to address these fundamental issues regarding conceptual clarity, methodological transparency, and internal consistency. The study has valuable data, and these revisions will ensure its message is communicated with precision and rigor**
