# Supplementary material for: Prevalence and risk factors of disordered eating risk among Yemeni medical students: A national multicenter cross-sectional study
Source: PLoS One. 2026 Mar 25;21(3):e0345771. doi: 10.1371/journal.pone.0345771 (PMC13016294; doi:10.1371/journal.pone.0345771)
Supplement: Attachment — Submitted filename: Point to point response.docx [file pone.0345771.s006.docx]

**Response to the Academic Editor and Reviewer #2**

Dear Dr. Zawiah and Reviewer #2,

We sincerely thank you for your careful review of our manuscript and for the constructive and insightful comments. We have revised the manuscript thoroughly to address all concerns raised. Below, we provide a detailed, point-by-point response outlining the revisions made.

**Response to the Academic Editor**

**Comment 1:**

The manuscript addresses an important topic and has a valuable national multicenter scope; however, several issues require clarification to improve rigor, consistency, and alignment of conclusions with the cross-sectional design.

**Response:**

We thank the Editor for this thoughtful assessment. We have carefully revised the manuscript to improve methodological transparency, internal consistency, and alignment of interpretation with the cross-sectional design. In particular, we have clarified sampling procedures, self-reported measures, variable selection criteria, and have moderated the tone of the Discussion and Conclusion to avoid causal language.

**Abstract**

**Comment 2:**

The proportion reporting a prior eating disorder diagnosis (31.6%) appears unusually high and exceeds the EAT-26 positive screening proportion (30.4%); clarify how “prior diagnosis” was defined/ascertained (self-report vs. verified) and ensure it is plausible and interpretable.

**Response:**

We appreciate this important observation. We have clarified in both the Methods and Abstract that prior diagnosis was assessed through a single **self-reported item** asking participants whether they had ever received a diagnosis from a healthcare professional, and that this information was not independently verified.

Additionally, we have added explanatory text in the Discussion to clarify that the discrepancy between lifetime self-reported diagnosis (31.6%) and current screening positivity (30.4%) may reflect symptom remission, differences between lifetime and current status, or potential misclassification inherent to self-report data.

**Methods**

**Comment 3:**

The recruitment is described as “official student communication platforms,” but there is no clear sampling method, no denominator, and no response rate.

**Response:**

We have added a dedicated **“Sampling and recruitment”** subsection to the Methods. We now clearly state that a **non-probability convenience sampling strategy** was used and that participation was voluntary via online distribution channels.

We have clarified that, due to distribution across multiple institutional and student-managed platforms, the exact number of students who received the survey invitation could not be determined; therefore, a response rate could not be calculated. We have also acknowledged this as a limitation in the Discussion.

**Comment 4:**

Selecting variables by bivariate p < 0.20 should be justified and consider including a priori confounders.

**Response:**

We have revised the Statistical Analysis section to justify the use of p < 0.20 as a preliminary inclusion threshold to avoid excluding potentially important confounders. We also clarified that key sociodemographic variables (e.g., age and gender) were retained in the multivariable model regardless of statistical significance due to theoretical relevance.

**Comment 5:**

“Descriptive cross-sectional study with an analytical component” is redundant.

**Response:**

We have revised the wording throughout the manuscript to state simply:
**“national multicenter cross-sectional study.”**

**Comment 6:**

Clarify EAT-26 validation and translation.

**Response:**

We have specified that both Arabic and English versions of the EAT-26 used in this study were previously validated and have now cited the relevant validation studies. We also clarified that official validated translations were used.

**Comment 7:**

Height/weight are self-reported; acknowledge measurement bias and specify BMI calculation and handling of implausible values.

**Response:**

We have clarified in the Methods that BMI was calculated using self-reported height and weight (kg/m²). We also added that implausible anthropometric values were screened during data cleaning and excluded based on biologically plausible ranges. This potential measurement bias is now explicitly acknowledged in the Limitations section.

**Results**

**Comment 8:**

The prevalence of prior diagnosis appears unusually high and there is a denominator mismatch (n=1,117 used twice).

**Response:**

We have carefully reviewed and corrected the reporting to eliminate any denominator inconsistencies. The figures for prior diagnosis and symptom reporting are now clearly distinguished and internally consistent. We have also clarified that prior diagnosis was self-reported and not verified.

**Comment 9:**

Psychiatric diagnoses are self-reported but treated as clinical diagnoses.

**Response:**

We have revised the Methods, Results, and Discussion to consistently refer to these variables as **“self-reported history of…”** psychiatric conditions. We explicitly state that these were not clinically verified and were not assessed using diagnostic instruments.

**Comment 10:**

Behaviors such as vomiting and laxative use must be framed descriptively, with clear time frames.

**Response:**

We have revised Table 4 and its accompanying text to ensure accurate totals and percentages (correcting a summation error). We now describe these behaviors in neutral, descriptive language and clearly indicate that frequencies refer to reported behavioral occurrence categories within the survey time frame. All percentages now sum correctly to the total sample (N = 3,540).

**Conclusion**

**Comment 11:**

The conclusion is overly long and expansive; avoid overstatement and temper implementation recommendations.

**Response:**

We have substantially shortened and revised the Conclusion. We removed causal phrasing (e.g., “demonstrates,” “complex interplay”), softened recommendations (e.g., “consideration may be given to”), and aligned interpretation with the cross-sectional design. The novelty claim has been removed to avoid overstatement.

**Response to Reviewer #2**

We sincerely thank Reviewer #2 for the careful and constructive comments.

**Results – Table 1**

**Comment 1:**

The statement about sixth-year students is incorrect.

**Response:**

Thank you for identifying this. We have corrected the Results text to accurately state that **first-year students** had the highest proportion of disordered eating risk (23.2% vs. 14.7%). The incorrect reference to sixth-year students has been removed.

**Comment 2:**

Replace vague wording with explicit comparisons.

**Response:**

We have revised the Results section to include precise percentage comparisons throughout (e.g., “16.4% vs. 5.1%”), rather than general phrases such as “higher proportions were observed.”

**Comment 3:**

Highlight strongest associations (>3x higher prevalence).

**Response:**

We have revised the Results text to explicitly emphasize strong differences for suicide attempts, autism spectrum disorder, and type 1 diabetes mellitus.

**Comment 4:**

Indicate statistical significance in tables.

**Response:**

We have revised table formatting to clearly indicate statistically significant findings.

**Results – Figure 1**

**Comment 5:**

Replace “no significant weight” with descriptive wording.

**Response:**

We have corrected this to “no weight change” to avoid inappropriate statistical terminology in a descriptive figure.

**Results – Table 2**

**Comment 6:**

Include comparative percentages and correct inaccurate statement regarding borderline personality disorder.

**Response:**

We have revised the Results text to include explicit comparative percentages (e.g., PTSD 8.3% vs. 3.0%). We corrected the statement to reflect that borderline personality disorder was not statistically significant. Table footnotes now include abbreviations (e.g., PTSD, OCD).

**Results – Table 4**

**Comment 7:**

Total exceeded sample size.

**Response:**

We sincerely thank the reviewer for identifying this error. We have recalculated all frequencies to ensure each row sums exactly to N = 3,540 (100%). The table and corresponding text have been corrected accordingly.

**Discussion**

**Comment 8:**

Clarify marital status contradiction.

**Response:**

We have revised the Discussion to clarify that marital status was significant in bivariate analysis but was not retained as an independent predictor in the adjusted multivariable model.
